# Decision-Making System for the Diagnosis of Syndrome Based on Traditional Chinese Medicine Knowledge Graph

**DOI:** 10.1155/2022/8693937

**Published:** 2022-02-10

**Authors:** Rui Yang, Qing Ye, Chunlei Cheng, Suhua Zhang, Yong Lan, Jing Zou

**Affiliations:** School of Computer, Jiangxi University of Chinese Medicine, Nanchang 330004, Jiangxi, China

## Abstract

The clinical informatization of traditional Chinese medicine (TCM) focuses on serving users and assisting in diagnosis. The rules of clinical knowledge play an important role in improving the TCM informatization service. However, many rules are difficult to find because of the complexity of the data in the current TCM syndrome prediction. Therefore, we proposed an end-to-end model, called Decision-making System for the Diagnosis of Syndrome (DSDS), which is based on the knowledge graph (KG) of TCM. This paper introduces the link prediction for the diagnosis of syndrome by dismantling medical records into multiple symptoms. In addition, based on the symptoms and predicted syndromes, the most relevant syndrome could be determined by the scoring and voting method in this paper. The results show that the accuracy of DSDS is 80.6%.

## 1. Introduction

Knowledge graph (KG) is a multirelational graph composing of entities and relationships [1]. Recently, KG has attracted great attention from academia and industry. Many examples of large KGs include Freebase [2], DBPedia [3], Yago [4], and NELL [5]. Furthermore, decision-making systems on KG have become an important research area in the past few years. In medicine recommendation systems, the recommended syndrome is based on the analysis of KG, which contains a lot of Chinese medicine information. It is challenging to build the ontology of Chinese medicine and use data to build a KG to realize the application of downstream tasks. In the previous study, Yu et al. [6] constructed a KG of traditional Chinese medicine (TCM) health care based on the “TCM health care ontology” and formed a KG based on the semantic network and further developed retrieval, browsing, and visualization methods. Zhou et al. [7] used clinical data to build a warehouse, which combined structured electronic medical record (EMR) data for medical knowledge discovery and TCM clinical decision support. These studies have promoted the development of Chinese medicine informatization. Unfortunately, most previous studies have focused on the theoretical aspects of Chinese medicine, and there are not many studies in the field of syndrome recommendation.

In the TCM decision-making system, it is necessary to reason about four diagnosis methods and related information in the EMR and analyze the recommended syndromes [8]. This system based on the KG of TCM mainly relies on the intrinsic relationship between symptoms and syndromes. Many studies have proposed auxiliary diagnosis and treatment methods based on machine learning and deep learning [9]. These methods can discover rules in clinical prescriptions of TCM, including the potential connection between symptoms and syndromes [10, 11]. However, these methods lack the TCM dialectical thinking, and the problem of symptom overlap is difficult to solve.

**Figure 1:**
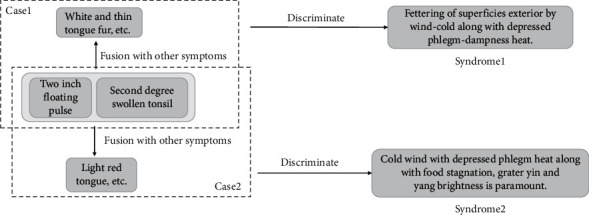
An indication of the overlapping of symptoms between syndromes.

We used the example in Figure 1 to illustrate this problem. For example, “two-inch floating pulse (两寸浮)” and “second-degree swollen tonsil (扁桃体二度肿)” were symptoms in EMRs, but both were included in “Syndrome1” and “Syndrome2.” Thus, it is not easy to identify syndrome through repeated signs. Therefore, we constructed a KG that retains the diagnostic information of a single medical record. The KG can maintain the relationship between the symptom information in syndromes. We generated the triples of the predicted medical records that lack the tail entity and completed triples to obtain the recommended syndromes from the medical records through the scoring and voting method.

We made the following contributions: (1) This paper constructed a KG based on EMR of TCM (KG-EMR-TCM). It contained a single EMR information of TCM. (2) Decision-making System for the Diagnosis of Syndrome (DSDS) can flexibly combine symptom information and reduce symptom overlap between syndromes. In addition, the accuracy of diagnosis reached 80.6% for five similar syndromes. (3) Even if the predicted result of DSDS is different from the label, there is a certain similarity in the symptoms with the expected symptom, expanding the diagnostic thinking.

## 2. Related Work

### 2.1. Knowledge Graph Completion Methods

In recent years, link prediction in the KG embedded by KG has become a hot field. The general framework defines a scoring function for triples (*h*, *l*, ?) in KG and constrains them so that the score of the correct triple is greater than that of the wrong triples [12].

TransE [13] embedded entities into a high-dimensional real space and relation as translation between the head and the tail entities. RESCAL [14] and DistMult [15] consist of a score function containing a bilinear product between the head entity and tail entity vectors and a relation matrix. ComplEx [16] represented entity vectors and relation matrix in the complex space. ConvE [17] used convolutional neural network to learn the scoring function among head entity, tail entity, and relationships. RotatE [18] projected entities in the complex space, and relations are represented as rotations in the complex plane. InteractE [19] added feature interaction based on ConvE. Conv-TransE [20] kept the global learning metric for entities and relation entities embedded in triples unchanged.

### 2.2. TCM Knowledge Graph and Its Applications

Li et al. [21] constructed a graph containing 16,217,270 deidentified clinical visit data. They proposed a novel quadruplet structure instead of the classical triplet in KG. TCMRel [22] constructed a candidate relation graph composed of four types of node: herbs, formula, syndrome, and disease, connected by five types of links: formula-disease, formula -syndrome, herbs-disease, herbs-syndrome, and disease-syndrome. CMD [23] used TCM information to create a four-node type graph and proposed a diagnosis model based only on symptoms according to the network. CLLT [24] contained three types of nodes to construct clinical maps: herb, symptom, and diseases and their correlations from 7,000 clinical prescriptions for lung tumors. Furthermore, the system can use the patient's current symptoms as input to obtain medical advice, possible diseases, and treatment options. Xie et al. [8] used ancient TCM books to construct a KG and inferred from symptoms to syndromes. This can assist in diagnosis and treatment. Balažević et al. [25] embedded node2vec into the existing KG and recommended prescription based on the similarity between the vectors. Trouillon et al. [26] proposed an improved algorithm to learn representation vectors from probabilistic medicine KG and applied embedding to link prediction.

Compared with the previous works on the application of TCM, there are several novelties in our work. The basic principle of TCM understanding and treatment of diseases is syndrome differentiation based on the holistic concept of TCM. We converted a complex dialectical problem into a simple completion problem of multiple symptoms in the KG. This is in line with the diagnosis of syndrome based on the four-diagnosis information. In addition, we evaluated the results of simple questions and obtained relevant syndromes.

## 3. Methods

### 3.1. Framework Overview

**Figure 2:**
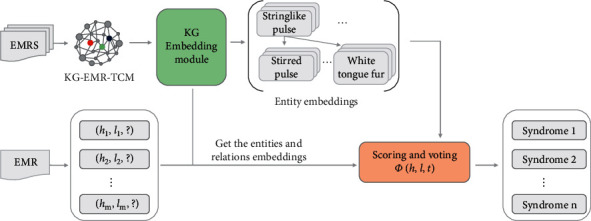
DSDS model architecture based on KG-EMR-TCM.

The DSDS in this paper was divided into three steps: KG-EMR-TCM construction module, KG-EMR-TCM representation module, and the syndrome prediction module. The framework of DSDS is shown in Figure 2.

**Step 1 .**

**Figure 3:**
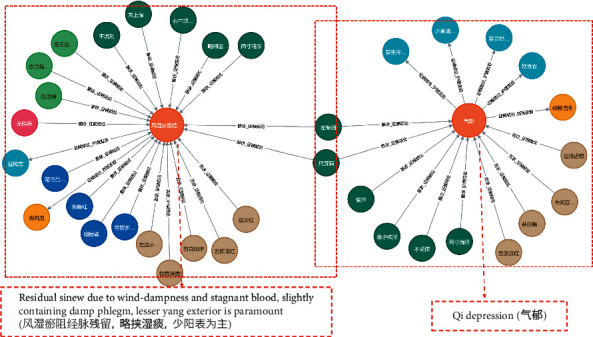
Display of two medical records structures in KG-EMR-TCM.

We processed the data and formulated logical structure of the symptom information and syndromes (Figure 3).

**Step 2 .**

Then, we used the processed data to build KG-EMR-TCM and created embeddings for all entities and relationships in KG.

**Step 3 .**

We completed missing triples by KG and then obtained top-N recommended syndromes through the scoring and voting module.

The detailed information about each module is in the following subsections.

### 3.2. KG-EMR-TCM Construction Module

There is a wealth of knowledge in Chinese medicine records, including symptoms, physical examination, diagnosis, treatment principles, and so on. After desensitizing the existing 131650 TCM medical records, 108746 medical records remain. We used the method of aggregating the information of a single EMR to construct a KG. In this way, the symptoms of a medical record can be effectively associated with the syndrome, which can be used to dig out the complex relationship between symptom and syndrome. The experiment of syndrome recommendation proved the validity of the KG construction. Figure 3 is an example of two medical records in KG. In this example, there are two symptoms of the two syndromes that are repeated, namely “the left side thin (左偏细)” and “the weak foot(尺沉弱).” Through the KG, we can find the associated symptoms between different syndromes.

According to the logical identifying symptom of TCM diagnosis, this paper constructed a KG and aggregated the entities in the KG. Figure 3 shows the two syndromes in KG-EMR-TCM with related entities. We used the EMRs to build KG-EMR-TCM and stored it in Neo4j.

### 3.3. KG-EMR-TCM Embedding

The KG embedding model embeds all entities and relationships into low-dimensional vectors and captures their semantics [27]. For each *e* ∈ *ℰ* and *l* ∈ ℒ, KG embeddings models generate *e*_*e*_ ∈ ℝ^*d*_*e*_^ and *e*_*r*_ ∈ ℝ^*d*_*r*_^, where *e*_*e*_ and *e*_*r*_ are *d*_*e*_ and *d*_*r*_ dimensional vectors, respectively. Each embedding method also has a scoring function *ϕ*(*·*):  ℰ × ℒ × *ℰ*⟶ℝ to assign score *ϕ*(*h*, *l*, *t*) to a possible triple (*h*, *l*, *t*)*h*, *l* ∈ *ℰ*, *l* ∈ ℒ. We trained in a certain way that for each correct triple (*h*, *l*, *t*) ∈ *KG* and wrong triple (*h*, *l*, *t*′) ∉ *KG*, the model assigns scores such that *ϕ*(*h*, *l*, *t*) > 0 and *ϕ*(*h*, *l*, *t*′) < 0. The scoring function is usually a function of (*e*_*h*_, *e*_*l*_, *e*_*t*_).

ComplEx model represented entities and relations in a complex space instead of using a real-valued space. We captured both symmetric and antisymmetric relations in this model and used the hermitian dot product to do composition for relation, head, and the conjugate of the tail. Given *h*, *t* ∈ *ℰ* and *l* ∈ ℒ, ComplEx generated *e*_*h*_, *e*_*l*_, *e*_*t*_ ∈ ℝ^*d*^a and defined the scoring function:

(1) ϕh,l,t=Reeh,el,e¯t=Re∑k=1dehkelke¯tk,

where *ϕ*(*h*, *l*, *t*) > 0 is for correct triples and *ϕ*(*h*, *l*, *t*) < 0 is for wrong triples. *Re* represents the genuine part of a complex number.

### 3.4. Syndrome Prediction

In the DSDS model, we used a series of complete triples for training, such as (pulse taking, performance of pulse taking, two-inch floating pulse) and (two-inch floating pulse, pulse taking-syndrome, fettering of superficies exterior by wind-cold along with depressed phlegm-dampness heat). However, when we tested an EMR, the syndrome was the predicted answer. Thus, we classified the test EMR according to the word segmentation rules and obtained a series of missing triples (*h*, *l*, ?), such as (two-inch floating pulse, pulse taking-syndrome, ?) and (white and thin tongue fur, tongue inspection-syndrome, ?). We used KG-EMR-TCM to predict these triples with missing tail entities. The tail entities of the missing triples are complemented by the score function of the ComplEx model. There was a corresponding score for each completed triple [25, 27].

The value obtained from the scoring function was meaningless. To interpret the score [16], we generated the corruptions and compared the triple score against the scores of corruptions. This shows how the model ranks the test triple against them. We chose the top-N of the rank as the correct option. Therefore, we were learning to rank task instead of classification task. If there are *m* symptoms, we defined (*h*, *l*, *t*') obtained by negative sampling as *T*_1_, *T*_2_,…, *T*_*m*_, and for each *T*_1_, *T*_2_,…, *T*_*m*_ candidate *n* entities, we used an *m* × *n* matrix to represent all candidate prediction syndromes.

**Figure 4:**
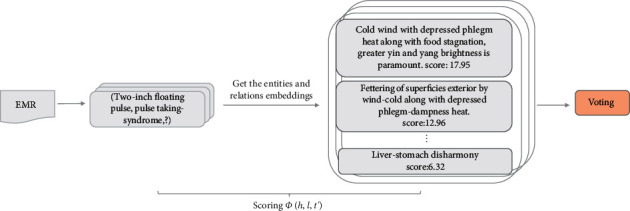
The scores for predicting syndromes.

After obtaining *T*_1_, *T*_2_,…, *T*_*m*_, their scores were calculated, as shown in Figure 4. The higher the score, the more suitable the combination of triples. However, due to the problem of symptom overlap between syndromes in TCM, the highest score did not mean that the current EMR was correct. Therefore, we proposed a scoring and voting method to solve this problem.

We counted all the candidate syndromes by using *f*(*·*) and then used *G*(*·*) to rank the syndromes with the highest number of occurrences. *f*(*·*) is the calculation method of the score function of complex, and *G*(*·*) is the method of frequency statistics. Finally, we obtained *S*_*n*_, which was the recommended syndromes.

(2) $$\begin{array}{c} S_{n}=G\left(\sum_{i=1}^{m}\sum_{j=1}^{n}f\left(T_{ij}\right)\right). \end{array}$$

The comprehensive judgment of all symptoms can solve the problems caused by multiple symptoms and comprehensively consider the impact of all symptoms.

## 4. Results

### 4.1. Dataset

The clinical medical records were mainly from QIHUANG TCM. After desensitization, each case in the medical record data provided the symptoms and related syndromes of the fourth diagnosis of TCM, body surface examination, diagnosis of TCM, and nursing precautions. After cleaning and formatting the database, 108,746 cases were finally obtained as functional syndromes, including many symptoms and syndromes of TCM. In order to discover more information between symptoms and syndromes, we performed statistics on entities in KG.  Table 1 shows that the average percentage of repeated symptoms in eight types of entities reached 91.2%. This indicates that the percentage of repeating symptoms between different syndromes was high, and the relationship was complicated.

Based on 202,619 entities, we constructed 1,499,457 triples in KG-EMR-TCM. Tongue inspection and pulse-taking entities were the most significant among the syndrome-related entities, and listening and smelling were the least significant. The repetition of nursing precautions, listening, and smelling in all medical records were the highest, ranging from 96.7% to 97.5%. The recurrences of these two types of entities in different syndromes were very high.

Most of the information in the medical records is relatively standardized, so this paper used punctuation to segment the four-diagnosis information, body surface examination, and nursing precautions and added the data after the segmentation to the database. In addition, the expression of Chinese medicine is complicated, and the establishment of the synonym table is much work, so this paper does not establish a synonym table.  Table 1 shows the statistics after entity processing. The columns in the list: the name of the entity type, the number of entities in the original data, the number of entities after word segmentation, the number of entities remaining after deduplication, and finally, the repetition rate of the entities. In order to maintain the richness of KG-EMR-TCM, we added nursing precautions when constructing the KG, but they were not used as the basis for diagnosis and decision-making when the actual symptoms were recommended.

### 4.2. Experimental Setup

There are few studies on predicting specific TCM syndromes, and most of them used expert systems. We adopted the methods of multiclassification and multilabel classification to measure the effectiveness of our model for the TCM clinical medical record in this paper.

In order to verify the performance of DSDS, we compared it with the following methods: GBC [28] is a one-vs-rest multiclassification strategy that fits a classifier to each category. XML-CNN [29], an algorithm for large-scale multilabeled data, is a classic method of convolutional neural networks for multilabel text classification. We used the maximum pooling layer to add as much feature information as possible. KG-XML-CNN added the KG-EMR-TCM vector in this paper based on XML-CNN for more input information to improve the accuracy.

The dataset used in the comparison experiment is KG-EMR-TCM. The difference is that GBC, KG-XML-CNN, and the DSDS all use KG-EMR-TCM embedding vectors as input, whereas XML-CNN uses KG-EMR-TCM randomly generated vectors as input.

### 4.3. Experimental Results

We conducted a series of comparative experiments on syndrome prediction to simulate dialectical clinical analysis. To obtain node embeddings, we selected a set of symptom and syndrome nodes and used their representations as feature to learn and test the application of syndrome prediction based on EMRs. Considering the uneven distribution of various categories of Chinese medicine data, we used accuracy, weight-recall, weight-f1 as metrics for evaluation. Different classes are given different weights through the weighted average method. The proper distribution ratio of the category determined the weights. Each type was multiplied by the weight and then added.

For the convenience, we have defined the following symbols. The samples in the studied class are positive samples, and the elements in other classes are negative samples. *TP*_*i*_ indicates that there is a correct positive sample classification in a certain category. *FP*_*i*_ indicates that a negative sample is misclassified as a positive sample in a certain class. *FN*_*i*_ indicates sample misclassified as a negative sample in a certain class.

Precision and recall calculation under each category are as follows:

(3) $$\begin{array}{c} Precision_{i}=\frac{TP_{i}}{TP_{i}+FP_{i}}, \end{array}$$

(4) $$\begin{array}{c} Recall_{i}=\frac{TP_{i}}{TP_{i}+\;FN_{i}}. \end{array}$$

*W*_*si*_ represents the weight of each category in the calculation, and *N*_*si*_ represents the true number of samples in that category.

(5) $$\begin{array}{c} W_{s1}:W_{s2}\ldots W_{si}=N_{s1}:N_{s2}\ldots N_{si}. \end{array}$$

After calculating *Precision*_*i*_ and *Recall*_*i*_, the following indicators are calculated:

(6) $$\begin{array}{c} Precison_{Weighted}=\frac{\sum_{i=1}^{L}Precision_{i}\times W_{i}}{\left|L\right|}, \end{array}$$

(7) $$\begin{array}{c} Recall_{Weighted}=\frac{\sum_{i=1}^{L}Recall_{i}\times W_{i}}{\left|L\right|}, \end{array}$$

(8) $$\begin{array}{c} F1_{Weighted}=\frac{2\times \;Precison_{Weighted}\;\times \;Recall_{Weighted}}{Precison_{Weighted}\;+\;Recall_{Weighted}}. \end{array}$$

After we have calculated the above indicators, we need to measure the accuracy of the DSDS model for the prediction of syndrome results. In equation (9), *pre* is the number of correct cases for the predicted syndrome, and *Total* is the total number of tested medical records.

(9) $$\begin{array}{c} Accuracy=\frac{pre}{Total}. \end{array}$$

We design an end-to-end model; so, the following method is used to evaluate the accuracy of the model calculation: when the syndrome predicted by the model is the same as the label, it is correct; when the accuracy under the candidate top-N is correct, the label of the test case appears in the *n* results predicted by the model.

The results obtained by equations (3)–(9) are shown in Table 2.

**Figure 5:**
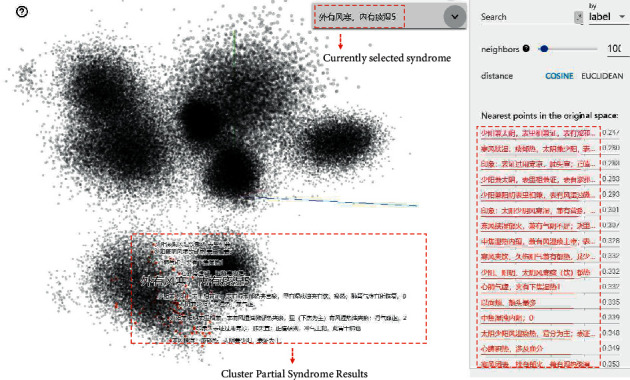
Analysis of KG-EMR-TCM embedded clustering.

We used the syndrome groups (Figure 5) as candidate sets for the clinical syndrome prediction to reduce the number of syndromes predicted. The performance of all the methods on TCM dataset are listed in Table 2. DSDS showed better performance than GBC and XML-CNN in all metrics. The overall performance of the traditional machine learning model based on GBC was worse than that of the method based on deep learning.

In Table 2, there are no results for the GBC model. The first reason is that the GBC model obtained the symptom vectors in KG-EMR-TCM as a feature for training. However, the repetition among the facts in Table 1 reached 91.2%. This means that the vectors trained in the GBC model had a certain similarity. The second reason is that the experiment can obtain valid metrics to evaluate when the number of samples and categories was reduced. However, the metrics values were too small to compare.

When the vector representation of symptoms and syndromes were added to the model XML-CNN, accuracy, weight-recall, and weight-f1 of KG-XML-CNN were 5.2%, 5.9%, and 5.3% greater than XML-CNN, respectively. This proves that the vectors of syndrome-related entities and relationships obtained after embedding KG-EMR-TCM through the representation learning model for KG were better than directly randomly generating the vector of input syndrome-related entities and relationships.

Finally, DSDS was compared to KG-XML-CNN, although KG-XML-CNN was slightly better on accuracy, DSDS was 3.4% and 3.6% higher than KG-XML-CNN on weight-recall and weight-f1, respectively. The results show that the improved multiclass DSDS model based on the ranking has particular effectiveness. The central part of the DSDS superior to KG-XML-CNN is that the DSDS disassembles the input triples, successfully distinguishes the symptom-related symptoms, and casts a vote on the symptom prediction to give the predicted syndrome.

### 4.4. Decision-Making Performance

Chinese medicine auxiliary decision-making aims to provide doctors with top-N recommended syndromes to facilitate follow-up treatment. The recommended syndromes ranked 1, 3, and 5 of this experiment are listed in Table 3. When only one recommended syndrome was given, the accuracy reached 62.1%. When three recommended syndromes were given, the accuracy was increased by 8.9%. When five recommended syndromes were given, the accuracy was 9.6% greater than that of top-3.

This paper aims to rank node pairs in terms of their relevancy, leading to potential linkages between syndromes. This potential linkage indicates that even if the predictive syndrome is incorrect, there are some symptoms overlap between the predicted syndrome and the right syndrome. This is conducive to expanding diagnostic thinking.

When the number of the recommended syndrome is one, we have given an analysis for the wrong predicted syndrome. In Table 4, the right syndrome is “fettering of superficies exterior by wind-cold along with depressed phlegm-dampness heat (风寒闭表,兼有痰湿郁热),” and the prediction is “cold wind with depressed phlegm heat along with food stagnation, greater yin, and yang brightness is paramount (寒风挟痰郁热, 兼有食滞, 太阴阳明为主).”

There was an inevitable overlap in the related entities between the correct syndrome and the predicted syndrome, such as “two-inch floating pulse” and “second-degree swelling of tonsils.” However, the scores of “stringlike pulse” and “cough” were lower than other entities. This indicates that they were more closely related to other syndromes.

Furthermore, we compared the following two scores: (1) the score was calculated to predict the syndrome and its symptoms and (2) the score between the predicted symptoms and the correct syndrome was calculated. From the data in Table 4, the latter's scores were lower than those of the former, and the symptoms with higher scores of the latter are related entities that overlap with the correct syndrome. This indicates that there is a certain similarity between the predicted syndrome and the correct syndrome. Therefore, even if the predicted syndrome is different from the correct syndrome, doctors can get help from DSDS.

### 4.5. Representation of KG-EMR-TCM

After constructing the KG, we embedded the complex vector space through the ComplEx model. The comparison between XML-CNN and KG-XML-CNN proved the effectiveness of the embedding.

In order to further study the embedding effect of KG-EMR-TCM, we analyzed the embeddings through TensorBoard. In Figure 5, all entities had successfully been gathered into eight categories. These corresponded to the conclusions of pulse taking, tongue inspection, listening and smelling, inspection, body surface examination, diagnosis of TCM, nursing precautions, and syndrome. In addition, the distances between the nodes of the syndrome type are the nearest points in the original space, listed in the right column of Figure 5.

## 5. Conclusion

In this paper, we constructed a KG based on clinical TCM data and proposed a syndrome prediction model. DSDS can realize the recommendation of syndromes through KG representation learning method. Experimental results show that the performance of DSDS in syndrome prediction was significantly better. This proved the effectiveness of DSDS. When the number of recommended syndromes was five, the accuracy of predicting syndromes reached 80.6%. This proved the effectiveness of DSDS. The model can perform auxiliary diagnosis, and the recommended syndromes are similar to a certain degree.

## Figures and Tables

**Table 1:** Statistics of each entity category in KG-EMR-TCM.

Entity type	Original	Participle	Deduplication	Percentage of repeating (%)
Tongue inspection	121709	371760	34144	90.8
Pulse taking	121545	462192	39633	91.5
Listening and smelling	7972	116435	2899	**97.5**
Inspection	33758	152316	12911	91.5
Body surface examination	63248	248111	28752	88.4
Nursing precautions	57288	315496	10262	**96.7**
Diagnosis of TCM	100038	95543	12102	87.3
**Syndrome**	108746	—	61916	85.8
Total	614304	1870599	**202619**	**91.2**

Bold shows the target predicted by this article, the total number of entities, categories with the highest repetition rate, and overall repetition rate.

**Table 2:** Performance comparison of different models.

Method	Weight-recall	Weight-*F*1	Accuracy
GBC	—	—	—
XML-CNN	0.525	0.527	0.574
KG-XML-CNN	0.584	0.580	**0.626**
DSDS	**0.618**	**0.612**	0.621

Bold values represent the best results in the comparative experiment.

**Table 3:** Syndrome recommendation results.

Results of top-N	Accuracy
Top-1	0.621
Top-3	0.71
Top-5	0.806

**Table 4:** Comparison of symptom entity scores of syndromes.

Syndrome	Symptom		Score	Compare to true syndrome
	English	Chinese		
Fettering of superficies exterior by wind-cold along with depressed phlegm-dampness heat (true) 风寒闭表,兼有痰湿郁热	Stringlike pulse	脉偏弦	4.40	—
	**Two-inch floating pulse**	**两寸浮**	**12.96**	—
	Reddish tongue texture	舌质偏红	9.65	—
	White and thin tongue fur	苔薄白	9.00	—
	The middle and back part of the tongue is thicker	中后部略厚	8.95	—
	**Second-degree swollen tonsil**	**扁桃体2度肿**	**9.34**	—
	Cough	咳嗽	4.40	—
Cold wind with depressed phlegm heat along with food stagnation, greater yin, and yang brightness is paramount (predict) 寒风挟痰郁热,兼有食滞,太阴阳明为主	Little stringlike pulse	脉略弦	10.98	9.56
	Stirred pulse	不静	16.53	9.30
	**Two-inch floating pulse**	**两寸浮**	17.95	**12.96**
	Light red tongue	舌质淡红	9.62	6.02
	White tongue fur	舌苔白	13.55	3.05
	**Reddish pharynx Larynx**	**咽喉略红**	14.70	8.37
	**Second-degree Swollen tonsil**	**扁桃体2度肿**	14.19	**9.34**
	Phlegm rale in the lung	两肺闻及痰鸣音	7.53	2.10
	Common cold	感冒	3.95	0.03

Bold values show the values mentioned in the article.

## Data Availability

The data used to support the findings of this study are available from the corresponding author upon request.

## Abbreviations

EMR: electronic medical record
TCM: traditional Chinese medicine
DSDS: Decision-making System for the Diagnosis of Syndrome
KG: knowledge graph
KG-EMR-TCM: KG based on EMR of TCM.
